# The complete mitochondrial genome of the white-rot fungus *Phanerochaete sordida* YK-624

**DOI:** 10.1080/23802359.2022.2124830

**Published:** 2022-09-27

**Authors:** Toshio Mori, Hideo Dohra, Hirokazu Kawagishi, Hirofumi Hirai

**Affiliations:** a Faculty of Agriculture, Shizuoka University, Shizuoka, Japan; b Research Institute of Green Science and Technology, Shizuoka University, Shizuoka, Japan; c Graduate School of Science and Technology, Shizuoka University, Shizuoka, Japan

**Keywords:** Ligninolytic fungi, mitochondrial genome, *Phanerochaete sordida*, white-rot fungus

## Abstract

The white-rot fungus *Phanerochaete sordida* (Karsten) Eriksson and Ryvarden 1978 is known for its excellent ligninolytic activity and capability to degrade various recalcitrant organic pollutants. In this study, we determined the complete mitochondrial genome sequence of *P. sordida* YK-624. The mitochondrial genome is 129,567 bp in length with a GC content of 28.9%, and contains two ribosomal RNA genes, 26 transfer RNA genes, and 50 open reading frames, including 14 conserved proteins. Phylogenetic analysis based on the mitochondrial genome confirmed that *P. sordida* belongs to the family Phanerochaetaceae in the order Polyporales, and showed the general phylogenetic relationships.

## Introduction

White-rot fungi are a type of wood-decay fungi that can degrade lignin in wood. *Phanerochaete sordida* YK-624 (ATCC 90872) collected in Yakushima, Kagoshima, Japan (30°18′17″N, 130°34′30″E) is a typical saprotrophic white-rot basidiomycetous fungus belonging to the family Phanerochaetaceae that shows excellent lignin-degrading activity (Hirai et al. 1994). The fungus has been reported to have biodegradative activity for various organic pollutants, such as neonicotinoid insecticides (Mori et al. 2017; Wang et al. 2019). Among Phanerochaetaceae fungi, the mitochondrial DNA (mtDNA) sequence of only *Phanerochaete carnosa* has been available on GenBank to date. Although fungi belonging to the genus *Phanerochaete* are among the most intensively studied white-rot fungi, little information is available on their genome. Therefore, we newly determined the mtDNA sequence of *P. sordida,* and analyzed its phylogenetic relationships with other basidiomycetes. The mitogenome data provided in the present study will be useful for the investigation of detailed phylogenetic relationships of Phanerochaetaceae fungi.

## Materials and methods

*P. sordida* YK-624 which was isolated from decaying wood collected from Yakushima, Japan, was deposited at American Type Culture Collection (ATCC; https://www.atcc.org/, tech@atcc.org) as the voucher number ATCC 90872. The genomic DNA of this fungus was extracted using Isoplant II (Nippon Gene Co., Ltd., Tokyo, Japan). Purified genomic DNA by ethanol precipitation was fragmented by using a Focused-ultrasonicator M220 (Covaris Inc., Woburn, MA), then the genomic library was prepared using a TruSeq DNA PCR-free Library Preparation Kit (Illumina, San Diego, CA), and sequenced using MiSeq (Illumina, San Diego, CA) (Mori et al. 2021). Raw sequence reads (DRR311115 and DRR311116) were cleaned using Trimmomatic ver. 0.38 (Bolger et al. 2014) as described previously (Mori et al. 2021), and the resulting 8,146,783 read pairs totaling approximately 4,157 Mb were assembled using NOVOPlasty ver. 4.3.1 (Dierckxsens et al. 2017) with the nucleotide sequence of the cytochrome c oxidase subunit 1 gene from *P. carnosa* (GenBank accession no. MT090080.1, nt. 1–29,351) as the seed sequence. The obtained circular mtDNA was 129,567 bp in length with a GC content of 28.9% (GenBank accession no. LC707859). DNA annotation was conducted using the MFannot tool (http://megasun.bch.umontreal.ca/cgi-bin/mfannot/mfannotInterface.pl), then manually curated. The ribosomal RNA genes were predicted by aligning the mtDNA sequences to those of *P. carnosa* (NC_057606). Subsequently, a phylogenetic analysis based on the 14 conserved mitochondrial proteins in 14 Agaricomycetes and an Ustilaginomycetes was performed. The concatenated amino acid sequences were aligned using MAFFT ver. 7.427 (Katoh and Standley 2013), and the phylogenetic tree was constructed by the neighbor-joining method (Saitou and Nei 1987).

## Results

**Figure 1.**
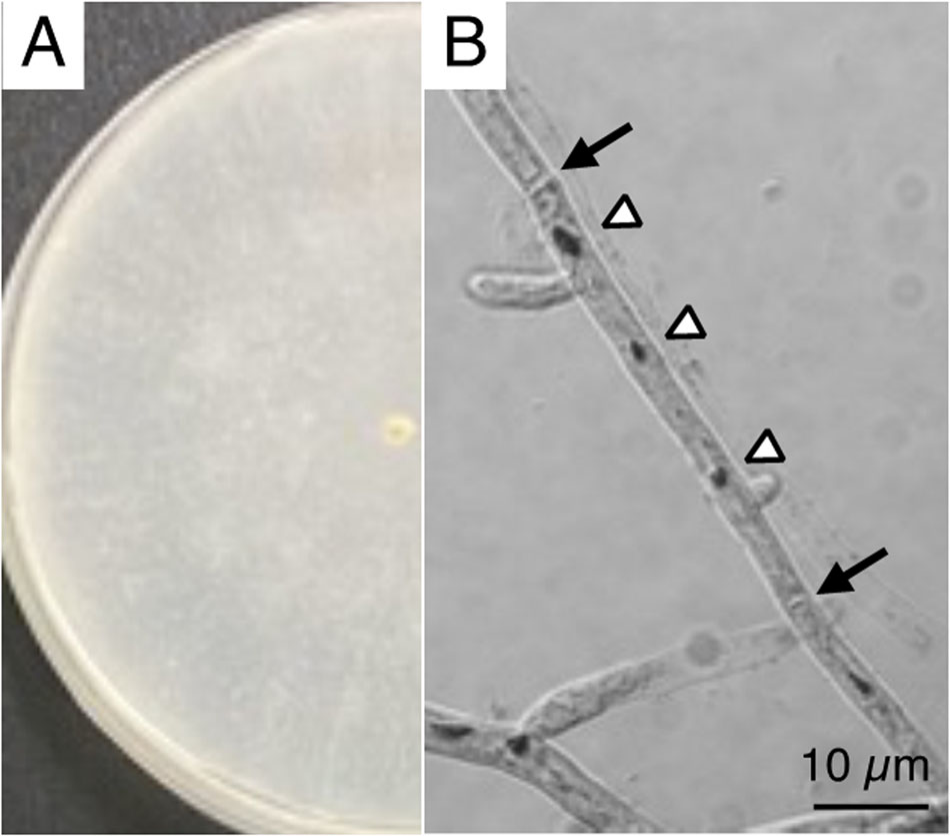
*P. sordida* YK-624 mycelium and hyphae observation. (A) Mycelia were grown on potato dextrose agar medium (i.d. 9 cm). (B) Hyphae were stained by HCl-Giemsa method following method described in previous study (Mori et al. 2020).

**Figure 2.**
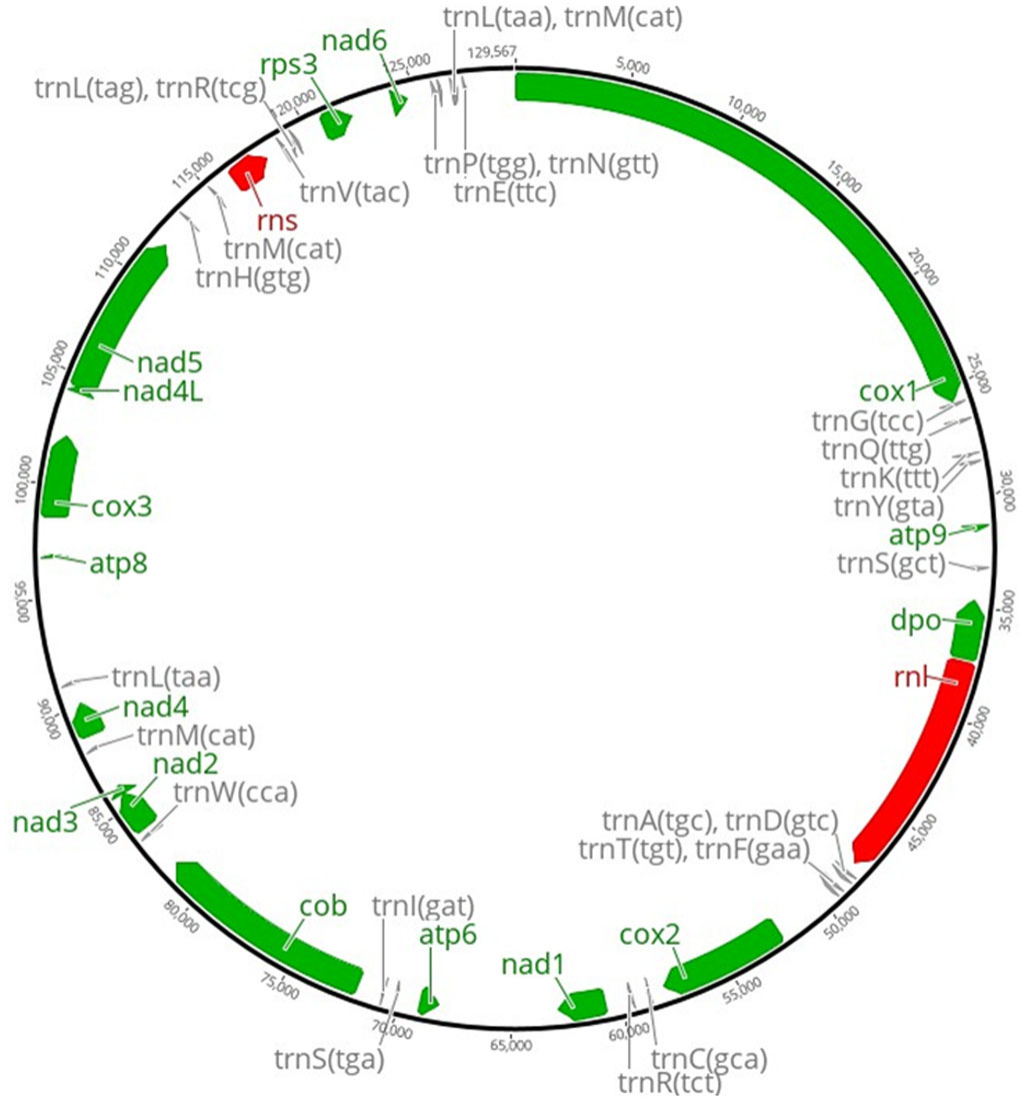
Mitochondrial DNA of *P. sordida* YK-624. The map of the 129,567 bp shows 14 conserved mitochondrial proteins (*cox1–3*, *cob*, *nad1–6*, *nad4L*, *atp6*, *atp8*, and *atp9*), DNA polymerase type B (*dpo*), ribosomal protein S3 (*rps3*), and tRNAs. Other 34 hypothetical proteins are not shown.

**Figure 3.**
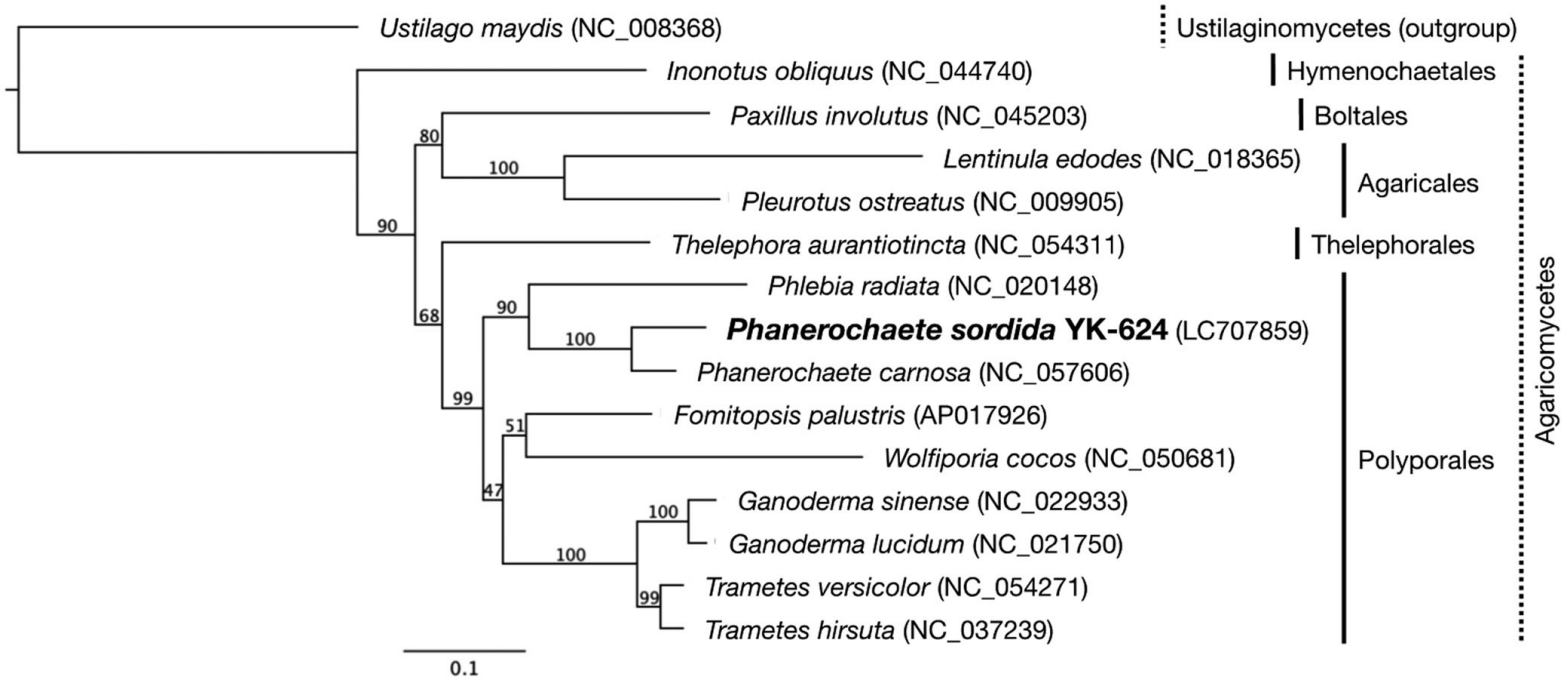
Phylogenetic relationships among 14 Agaricomycetes and an Ustilaginomycetes. The phylogenetic analysis was conducted by the neighbor-joining method based on the concatenated amino acid sequences of 14 conserved protein-coding genes (*atp6, atp8, atp9, cob, cox1–3, nad1–6,* and *nad4L*). *Ustilago maydis* (NC_008368) served as an outgroup. The bootstrap support values are shown at each node. The scale bar indicates the number of substitutions per site.

A white-rot basidiomycetes fungi *P. sordida* YK-624 forms white-colored filamentous fungi, and exhibits multi-nuclei hyphae without clamp connections (Figure 1) (Mori et al. 2020). Overall, 78 genes, including 50 putative protein-coding genes, two ribosomal RNAs (rnl and rns), and 26 tRNAs (for all 20 standard amino acids) were predicted in the mtDNA of *P. sordida* YK-624 (Figure 2). The 50 protein-coding genes encoded 14 conserved mitochondrial proteins (*cox1–3*, *cob*, *nad1–6*, *nad4L*, *atp6*, *atp8*, and *atp9*), DNA polymerase type B (*dpo*), ribosomal protein S3 (*rps3*), and 34 hypothetical proteins, most of which showed similarities to LAGLIDADG homing endonuclease. The phylogenetic tree showed that *P. sordida* is closely related to *P. carnosa* belonging to the family Phanerochaetaceae in the order Polyporales (Figure 3). The phylogenetic relationships between the mtDNA of *P. sordida* and other Agaricomycetes fungi are consistent with those based on nuclear genes.

## Discussion

In the present study, complete mitochondrial genome sequence of *P. sordida* YK-624 was newly determined. Although Phanerochaetaceae family included well studied groups like *Phanerochaete* and *Bjerkandera* fungi, classification study is not sufficiently progressed. In particular, the genus *Phanerochaete* is often very similar in morphology, and ITS (internal transcribed sequences) in the closely related species, so classification between each species is still uncertain (Floudas and Hibbett 2015). Although mitochondrial genome information on the genus *Phanerochaete* is currently very limited, the present study will assist in future taxonomic studies using mitochondrial genome information.

## Ethics statement

Ethical approval does not apply to this paper because the research material is mushrooms (fungus), and not human, animals, plants, or threatened/endangered species that require ethical approval.

## Author contributions

Conceived and designed the research: TM and HH. Performed the experiments and data analysis: TM and HD. Wrote the manuscript: TM and HH. Contributed to data interpretation, discussions, and critically reviewed the manuscript: HK. All authors reviewed the manuscript and approved the final version to be published.

## Data Availability

The genome sequence data that support the findings of this study are openly available in GenBank of NCBI (https://www.ncbi.nlm.nih.gov) under the accession number LC707859. The associated BioProject, SRA, and BioSample accession numbers are PRJDB11801, DRR311115, DRR311116, and SAMD00328051, respectively.

## References

[CIT0001] Bolger AM, Lohse M, Usadel B. 2014. Genome analysis Trimmomatic: a flexible trimmer for Illumina sequence data. Bioinformatics. 30(15):2114–2120. 24695404 10.1093/bioinformatics/btu170 PMC4103590

[CIT0002] Dierckxsens N, Mardulyn P, Smits G. 2017. NOVOPlasty: de novo assembly of organelle genomes from whole genome data. Nucleic Acids Res. 45(4):e18. 28204566 10.1093/nar/gkw955 PMC5389512

[CIT0003] Floudas D, Hibbett DS. 2015. Revisiting the taxonomy of *Phanerochaete* (Polyporales, Basidiomycota) using a four gene dataset and extensive ITS sampling. Fungal Biol. 119(8):679–719. 26228559 10.1016/j.funbio.2015.04.003

[CIT0004] Hirai H, Kondo R, Sakai K. 1994. Screening of lignin-degrading fungi and their ligninolytic enzyme activities during biological bleaching of kraft pulp. Mokuzai Gakkaishi. 40(9):980–986.

[CIT0005] Katoh K, Standley DM. 2013. MAFFT multiple sequence alignment software version 7: improvements in performance and usability. Mol Biol Evol. 30(4):772–780. 23329690 10.1093/molbev/mst010 PMC3603318

[CIT0006] Mori T, Dohra H, Suzuki T, Kawagishi H, Hirai H. 2021. Draft genome sequence of the white-rot fungus *Phanerochaete sordida* YK-624. Microbiol Resour Announc. 10(42):e0084221. 34672697 10.1128/MRA.00842-21 PMC8530024

[CIT0007] Mori T, Kondo O, Sumiya T, Kawagishi H, Hirai H. 2020. Self-fusion and fusion cell isolation of transformants derived from white rot fungus *Phanerochaete sordida* YK-624 simple visual method. J Biosci Bioeng. 129(2):146–149. 31506244 10.1016/j.jbiosc.2019.08.011

[CIT0008] Mori T, Wang J, Tanaka Y, Nagai K, Kawagishi H, Hirai H. 2017. Bioremediation of the neonicotinoid insecticide clothianidin by the white-rot fungus *Phanerochaete sordida*. J Hazard Mater. 321:586–590. 27694022 10.1016/j.jhazmat.2016.09.049

[CIT0009] Saitou N, Nei M. 1987. The neighbor-joining method: a new method for reconstructing phylogenetic trees. Mol Biol Evol. 4(4):406–425. 3447015 10.1093/oxfordjournals.molbev.a040454

[CIT0010] Wang J, Tanaka Y, Ohno H, Jia J, Mori T, Xiao T, Yan B, Kawagishi H, Hirai H. 2019. Biotransformation and detoxification of the neonicotinoid insecticides nitenpyram and dinotefuran by *Phanerochaete sordida* YK-624. Environ Pollut. 252:856–862. 31202138 10.1016/j.envpol.2019.06.022

